# How long is (too) long?

**DOI:** 10.1192/bjb.2019.24

**Published:** 2019-08

**Authors:** Birgit Völlm

**Affiliations:** 1 University of Rostock

## Abstract

Forensic psychiatric services care for patients who present with a mental disorder as well as a risk to themselves or others, and have usually been convicted of an offence. Their needs are complex and the length of stay (LoS) in forensic settings is long. LoS is affected by patient factors as well as legal and policy issues. Owing to the considerable economic and ethical issues surrounding lengthy stays in highly restrictive settings, it is crucial that a strategy is developed for how to deal with this patient group.

**Declaration of interest:**

None.

Earnshaw *et al*, in this issue, describe a study looking at the length of admissions in a medium secure unit in England over a period of nearly 30 years. They show a significant increase in length of stay (LoS) in the most recent cohort, as well as far more discharges to other psychiatric settings and fewer to independent living, with the diagnostic composition of the cohorts remaining largely unchanged. The paper is a welcome addition to the literature on LoS in forensic settings. Evidence on LoS is scarce, despite its obvious ethical and economic relevance. This editorial summarises the research on LoS to date and considers ideas to improve service organisation for this group of patients.

## Methodological issues

There are a number of ways to investigate LoS:^1^ (a) admission samples (i.e. considering all patients admitted during a particular period with LoS calculated from admission to discharge); (b) cross-sectional samples (the sample consisting of all patients resident in the particular unit of interest on a particular date with LoS calculated from date of admission to this time point); and (c) discharge samples (all patients discharged during a particular period with LoS calculated from date of admission to this date). Which method is the best depends on the questions to be answered. Earnshaw *et al* use admission samples, which has the advantage that the political and service provision context is likely to be the same for all patients in the sample at point of admission. What this method cannot capture is those patients who have not been discharged at the time of data collection, as their length of admission cannot be known at this point, thereby underestimating LoS. Another, and more significant, limitation of most research to date, regardless of which of the three approaches is used, is that it only considers a relatively short period in the patient's care trajectory, the admission to a single unit. In reality, patients' pathways are complex and an individual may be admitted to a number of secure units consecutively, adding to their overall LoS in secure care.

## What we know about LoS in forensic settings

Concerns that some patients stay for too long in too high a level of security were first raised following studies in high secure settings in the 1990s involving assessments by the patients' own teams as well as independent multi-disciplinary reviews. These suggested that between one-third and two-thirds of patients did not require that level of security.^2–5^ Inadequate provision of beds in less secure settings and inefficiencies in the system of transfer and discharge were thought to be significant factors in the delayed transfer to a more appropriate level of security. These findings led to the ‘accelerated discharge programme’,^6^ aimed at reducing patient numbers in high secure care while bed numbers in medium and low secure settings increased. At the beginning of the 1990s, there were 1700 high and 600 medium secure beds,^7^ while in 2015 there were just under 800 high and about 3200 medium secure beds.^8^ Although the reduction of high secure beds is a welcome development, the increase in the overall number of patients detained in secure settings is worrying. In addition, while I am not aware of any published research in this area, anecdotally, restrictions have increased in medium secure settings, e.g. with regards to leave and handcuffing during leave.

Maybe somewhat surprisingly, there is no agreement or guidance as to how long patients should stay in high secure settings in the UK. For medium secure care, the original guidance from government, based on the recommendations in the Glancy and Butler reports,^9^ suggested an upper limit of 2 years; however, a number of studies have demonstrated that this LoS is far exceeded in a large proportion of cases.^10^ In a multicentre study in the UK, including all three high secure hospitals and 23 medium secure services, both within the National Health Service and the independent sector,^11^ we found that 23.5% of high secure and 18.1% of medium secure patients fulfilled our criteria for ‘long-stay’. We defined ‘long-stay’ as having been in a high secure setting for more than 10 years, in a medium secure setting for more than 5 years or in a combination of both for more than 15 years. These figures were based on pilot work showing that these thresholds would identify a population large enough in size to provide meaningful conclusions for service developments, but not so large that a substantial proportion of the total patient population would be captured. Whether there has been an actual increase in LoS, however, remains unclear – the paper in this issue of the *Bulletin* is the first to investigate this question.

Research identifying factors associated with long stay is limited. In the UK, one early study at one of the three high secure hospitals^12^ identified severity of index offence as the most important factor for personality disordered patients, while for those with mental illness psychopathology was a more relevant predictor of LoS. Studies in medium secure settings have identified severity of psychopathology, psychiatric history, seriousness of offending, patients being on ‘restriction orders’ (requiring Ministry of Justice permission for transfer), non-engagement in interventions, dependency needs and lack of step-down facilities as factors associated with long stay. A review of the international literature^1^ similarly found that the factors most frequently associated with longer stay were seriousness of index offence, longer previous prison sentence, psychotic illness, symptom severity and having no close relationship.

Patient perspective has thus far been largely neglected in research on long stay in forensic settings. A qualitative study which formed part of the multicentre study described above^12^ and included 40 patient interviews investigated patients' perspective on reasons for long stay, their current situation and the prospect of moving on. Based on the emerging themes – attribution, outlook, approach and readiness for change – four overall ‘stances’ could be identified. Patients in the ‘dynamic acceptance’ group attributed their long-stay to themselves; they felt overall positive about therapy and being in secure care but felt they were ready to move on. Patients in the group we labelled ‘static acceptance’ attributed the reason for long stay internally and externally, were somewhat less positive about therapies and did not believe they were ready to move on. Those in the ‘dynamic resistance’ and ‘static resistance’ categories attributed their long-stay to external factors and were largely negative about their placement and interventions. Whereas the former group still believed they would move on eventually, the latter had largely given up on the prospect of moving on, despite their belief that they did not need to be in secure care.

## Service provision

Service provision in secure care is complex, entailing different levels of security with vague entry and even vaguer exit criteria. For example, those admitted to high secure care should present a ‘grave and immediate’ danger, obviously words that leave a lot of room for interpretation. Maybe somewhat surprisingly there is no agreement that those having entered high secure care presenting such a danger should then move on or be discharged if they no longer do so. In addition, how does one measure progress, e.g. of a patient having committed sexual offences against children? Such a patient might be very well adapted in any setting not giving access to children, but what should be the criteria to decide which level of security is the right one and when to move on after years of settled behaviour? The debate around the poor to moderate accuracy of risk assessment instruments for long-term predictions is also pertinent here.^13^ Unfortunately, in the UK there seems to be little appetite to tackle these complex questions nationally. Instead, each responsible clinician makes their own judgement, and in many cases has to fight individual battles with the next unit, trying to ‘sell’ their patient.

Little is known about the complex pathways forensic patients take. In theory, they move from higher to lower levels of security in accordance with the lowering of their risk and progress in therapy. In practice, such ideal pathways are rarely achieved. For instance, we showed in our study^11^ that less than one-third of the sample of long-stayers had stayed in their current secure unit only, while about 40% had stayed in three or more settings. More than 50% of long-stay medium secure patients had been admitted from another medium secure unit. This may be good practice in order to try a different approach in individuals with limited treatment gains. Nevertheless, it is clear that rather than moving *on*, a large number of patients seem to be moving *around*. It is difficult to see how this unfortunate state of affairs could be changed without taking a longitudinal view and without the development of national policy for this patient group.

Considering their pathways, the group of long-stay patients probably consists of three subgroups: (a) those who are still on a trajectory of positive, albeit slow, progress; (b) those who are ‘stuck’ currently but might move to less secure conditions under certain circumstances; and (c) those who require secure care for life. The first group is of least concern. The second might benefit from improvements in service organisation and advancements in psychotherapeutic and pharmacological therapies. The third group is most controversial. In our own study, consultants predicted that more than 40% of long-stay patients currently resident in high secure care would still be there in 5 years' time. Even for long-stay patients in medium secure care at the time of the study, only a minority of patients were expected to achieve independent living in the next 5 years.

Nevertheless, interviews with professionals in the UK demonstrated that staff working in secure units still conceptualise the process of care along the lines of ‘admission, treatment, rehabilitation, cure’, in denial of the actual situation of most patients.^14^ Staff felt uncomfortable with the idea of dedicated ‘long-stay units’, which they saw as warehousing. Many did not consider long periods of detention to be problematic as long as treatment was still offered, despite the fact that such treatment did not seem to make a difference to the patient's chances of moving on. Although these sentiments are understandable, not openly recognising long stay as a problem is likely to act as a barrier to considering service improvements for this patient group.

## International perspective

A number of countries have started to recognise the problem of long stay in forensic psychiatric hospitals, resulting in a range of legal and service provision developments.^15^ Croatia, Italy and Portugal now have legal provisions such that detention in hospital can no longer exceed the length of a prison sentence the individual would have been given had they been convicted as a non-mentally disordered offender. While not going that far, in Germany the constitutional court ruled that the length of detention has to be proportionate to the index offence and that the longer detention lasts, the more the individual's right to freedom weighs in relation to the protection of the public. While this principle has long been established in the case law of the German constitutional court, the new Criminal Code additionally specifies that after 6 years of detention in a forensic psychiatric hospital, detention has to be terminated unless there is a risk that further offences will be committed that will cause ‘serious’ physical or psychological harm to a victim; after 10 years such risk has to be ‘grave’.^16^

Other countries have developed policies and services specifically for long-stay forensic populations. One example of particular interest is service provision in The Netherlands. There patients can be given ‘long-stay status’ by a court on the application of their treating team. Criteria for this status are:

• having been an in-patient in a forensic institution for at least 6 years;
• having been a patient in two separate forensic hospitals;
• having completed relevant treatment programmes but with little discernible progress (or consistently refusing to participate in such programmes);
• no reduction in risk in the foreseeable future expected.

Individuals with long-stay status are diverted to specific long-stay units, where the emphasis is on quality of life rather than risk-reducing interventions. Crucially, an open discussion is held with the patient about this process and they are fully aware of their new status. Importantly, from a human rights point of view, this status is not a dead end; rather, patients can move back into mainstream provision if it is clinically indicated.

## Recommendations

Given the significant ethical and economic consequences of long stay in forensic care, it is essential that a national strategy is developed to deal with this complex patient group. Issues to consider in such a strategy are:

• taking a whole pathway approach;
• clear entry and exit criteria for services;
• cut-off points for the definition of ‘long stay’ in the different levels of security;
• independent reviews of long-stay patients;
• exploration of interventions designed to reduce LoS;
• improvement of the efficiency of pathways for this group;
• incentives to move patients on (e.g. through the Commissioning for Quality and Innovation framework, as is already happening in some trusts);
• flexibility in moving between services with prolonged transition periods;
• introduction and evaluation of pilot services for long-stay patients.

To develop such a strategy, wide consultation including patients and carers is required to capture relevant perspectives and concerns.

## About the author

**Birgit Völlm** is Professor of Forensic Psychiatry at the University of Rostock and Medical Director at the Hospital for Forensic Psychiatry, Rostock, Mecklenburg-Vorpommern, Germany.
